# Toward Environmental Sustainability, Health, and Equity: How the Psychological Characteristics of College Students Are Reflected in Understanding Sustainable Development Goals

**DOI:** 10.3390/ijerph18158217

**Published:** 2021-08-03

**Authors:** Meiai Chen, Eila Jeronen, Anming Wang

**Affiliations:** 1 School of Landscape Architecture, Zhejiang A & F University, Hangzhou 311300, China; 2 Department of Educational Sciences and Teacher Education, University of Oulu, FI-90014 Oulu, Finland; eila.jeronen@oulu.fi; 3 College of Materials, Chemistry and Chemical Engineering, Hangzhou Normal University, Hangzhou 311121, China

**Keywords:** sustainable development goals, attitudes toward sustainability, interest, motivation, self-efficacy, college students, psychological inventory

## Abstract

This study aimed to identify how the psychological characteristics of college students are reflected in understanding sustainable development goals (SDGs) by examining college students’ psychological characteristics, including attitude, interests, motivations, and self-efficacy, through the Sustainable Development Goals Psychological Inventory (SDGPI). The relationships among SDGs attitude, SDGs interest, SDGs motivation, and SDGs self-efficacy were analyzed by Pearson product-moment correlation coefficients. In addition, the Mann-Whitney U test and Kruskal-Wallis one-way analysis of variance were used to explore the differences among the college student groups in terms of gender, grade, and major in relation to attitude and personal characteristics. Attitude scores based on regression analysis were used to predict college students’ interest, motivation, and self-efficacy in relation to the SDGs. According to the results, (a) the college students considered the three most important SDGs to be good health and well-being (SDG 3) (49.72%), quality education (SDG 4) (41.39%), and no poverty (SDG 1) (32.22%), while the three least important SDGs were decent work and economic growth (SDG 8) (41.11%), partnerships for the goals (SDG 17) (38.06%), and response consumption and institutions (SDG 12) (30.83%); (b) the SDGPI had a high reliability, with a Cronbach’s alpha of 0.942; (c) there was a significant positive correlation between attitude and the variables of interest, motivation, and self-efficacy; (d) differences in attitudes, interest, and motivation between men and women were distinct and women scored much higher in these three subscales than men; (e) attitude could explain significant variance in interest, motivation and self-efficacy. In addition, attitude, interest, and motivation could account for self-efficacy. This study supports the development of sustainability education (SE) at the college level by providing new insights into college students’ psychological characteristics in relation to the SDGs.

## 1. Introduction

Sustainability has been considered a paradigm for thinking about the future in which environmental, societal, and economic considerations are balanced in the pursuit of an improved quality of life [1]. The ideals and principles behind this paradigm rest on the concepts of intergenerational equity, gender equity, social tolerance, poverty alleviation, environmental preservation and restoration, natural resource conservation, and building just and peaceful societies. Sustainable development is perceived as a socioeconomic system that enables human needs as well as long-term progress toward the well-being and improvement of overall quality of life in accordance with environmental constraints [2]. The United Nations Agenda 2030 for sustainable development includes 17 sustainable development goals (SDGs) [3]. The SDGs were produced in 2015 with an aim to eradicate poverty, protect the planet, and ensure prosperity for all. The COVID-19 pandemic may support efforts to implement the UN 2030 Agenda [4,5], as it has already influenced perceptions of the SDGs and more broadly research [6] and the actions of universities [7,8]. At the same time, the COVID-19 pandemic is slowing or undoing the fruits of global health and development [9], and it may jeopardize the process of the realization of the SDGs [10]. Eight of the 17 SDGs address the social dimensions of sustainable development and the relationship between them and the economic, environmental, and process dimensions. The COVID-19 pandemic makes it difficult to achieve the next social goals of sustainable development [11,12,13]: poverty reduction (SDG 1) [14,15], hunger eradication (SDG 2) [14], the promotion of good health and well-being (SDG 3) [16,17], decent work and economic growth (SDG 8) [18], and peace, justice, and strong institutions (SDG 16). It is also damaging education (SDG 4) [19] and undermining gender equality (SDG 5) and increasing inequalities (SDG 10). In addition, it impairs access to clean water and sanitation (SDG 6) [16,20].

**Figure 1:**
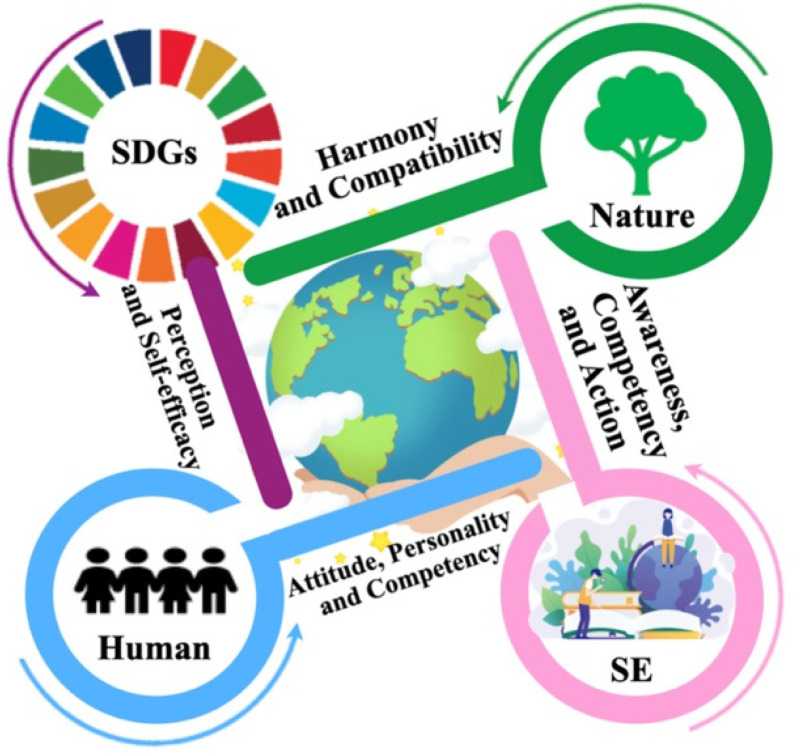
Scheme for investigating the importance of perception and self-efficacy with regard to the SDGs for the SE to advance the harmony and compatibility between humans and nature (own creation).

Education plays a central role in shaping individual and social change toward sustainability. In this study, we use the term sustainability education (SE) because it encompasses all forms of education for sustainable development [21]. SE transforms the research results of sustainability science into educational practices and guides the choice of learning objectives, relevant content, and appropriate learning and teaching methods [22]. Psychological characteristics are considered to be one of the cornerstones of effective teaching [23]; they encapsulate a complex set of attitudes, interests, motivations and self-efficacy [24,25]. There is currently a lack of research on the psychological characteristics of university students with respect to the SDGs. Previous research has shown that environmental attitudes are a powerful predictor of ecological behavior [26]. Interest in the environment has also been shown to have a significant effect on human behavior [27]. Motivation to protect the environment [28] and intrinsic motivation and self-efficacy also drive favorable environmental behavior [29]. Thus, from the perspective of teaching and learning processes, it is important to know how students’ psychological characteristics are reflected in their understanding of the SDGs. Based on the previous ideas, we conducted a survey expecting to fill an important research gap by investigating the attitudes, interest, motivation, and self-efficacy of Chinese college students with regard to the 17 SDGs (Figure 1) through the Sustainable Development Goals Psychological Inventory (SDGPI) [30]. 

## 2. Literature Review

### 2.1. The Psychology of Sustainability and Sustainable Development

Health, equity, and environmental sustainability can be improved through behavior and lifestyle changes [31,32,33], and supporting these changes is an important task for psychologists [34]. In the psychology of sustainability and sustainable development, sustainability is viewed both from the perspective of equity, ecological, and social environments [35,36] and from the perspective of promoting the well-being and quality of life of individuals [37,38,39,40]. While the traditional definition of sustainable development focuses on the preservation of natural resources, i.e., the avoidance of their exploitation, depletion, and irreversible alteration [41] the new definition focuses on the promotion of development, i.e., its diversification, growth, and flexible change [37,38,39,40]. Psychologists are committed to promoting people’s welfare while focusing on the issue of sustainability, so they should lead people to adopt a sustainable lifestyle [34]. Psychological processes are needed in decision-making on environmental issues, in guiding sustainable behavior, and in creating, promoting, and consolidating an environmentally sustainable culture. Psychology can be used to guide the creation of environments that maintain both physical and mental health and the sustainability of society [35]. Consequently, the psychology of sustainability and sustainable development can also provide new perspectives for the development of SE in universities. 

### 2.2. Four Psychological Characteristics Related to SDGs

Sustainability attitudes are a form of scientific literacy [42] that strongly indicate whether an individual will participate in scientific issues related to their personal life. These attitudes are also an indispensable part of sustainability consciousness. Studies show that individuals are more likely to report their own attitudes regarding direct experiences of the attitude object, and these attitudes are predictive of their future behavior [43]. Salas-Zapata and Cardona-Arias have studied university students’ knowledge of sustainable development and their attitudes and practices. Their study showed that these factors are interrelated, with higher levels of knowledge and attitudes than practices; furthermore, attitudes are related to gender and age [44]. According to Al-Naqbi and Alshannag, United Arab Emirates University students showed a high level of understanding, moderate positive behavior and very strong positive attitudes toward education for sustainable development and the environment [45], and female students were more knowledgeable about sustainable development/education for sustainable development than were male students. Leiserowitz, Kates and Parris found empirical trends in attitudes toward such values as freedom, democracy, equality, and globalization. They concluded that these values shape people’s willingness and ability to adopt the sustainability values, attitudes, and behavior needed to achieve global sustainability [46]. In addition to knowledge, interest in scientific issues has been found to have a partial and indirect effect on individuals’ sustainability attitudes [47].

SE in higher education is very important. Educators should consider which teaching and learning methods appear to be most effective in promoting a change in students’ sustainability attitudes [48]. Given that college students will become decision-makers for future sustainability issues, interdisciplinary curricula based on real-world sustainability challenges are needed to support students’ sustainability attitudes, skills, and competences [49]. Researchers from nine Italian universities assessed awareness, knowledge, and attitudes toward the SDGs and sustainability among first-year students. The results showed that students’ knowledge of the SDGs and Agenda 2030 was low, but their interest in SE and the SDGs was high in terms of both personal wisdom and the usefulness of professional knowledge [50]. Sustainability courses are often instrumental in the goal of communicating sustainability values and practices among university students. An example is a course on sustainable development called Engineering Sustainable Development, which was shown to have a positive impact on students’ beliefs, attitudes, and intentions about sustainable development [51]. Nousheen, Yousuf Zai, Waseem, and Khan investigated the impact of an education for sustainable development course on students’ attitudes toward sustainable development. Their results showed that attitudes were significantly improved as a result of the course [52]. 

In addition to attitude, motivation, interest, and self-efficacy have been studied in relation to sustainability. Previous environmental motivation studies have discussed the reasons why people who do not behave in an environmentally friendly way want to move to a more sustainable lifestyle. The motivation component relates to the behavior when actors consider reaction options for environmental change [53]. The results have shown that the main motivations for a more sustainable lifestyle are the desire to become more self-reliant, a desire for a healthier lifestyle, and the desire to avoid running out of resources [54]. 

Teachers’ didactic interest and self-efficacy predict their teaching practices, while students’ teaching interest and self-efficacy are significantly related to teachers’ interest in education and management goals [55]. It has been reported that self-efficacy significantly affects students’ sustainability behavior [56]. In a study of individuals’ sustainability awareness, knowledge, and responsibility in relation to the difficulty of the task and the efforts required, it was found that self-efficacy explained sustainability attitudes, knowledge, and the attitude-behavior gap [57]. A study of students’ motivation and attitudes showed that in addition to self-efficacy, threats to sustainable action are important predictors of behavioral intention [58]. Motivation and self-efficacy affect an individual’s action and performance, so they are of great importance in achieving the 17 SDGs. Another study [59] described a model that focused on the role of self-efficacy and belief in the variability of behavior in motivating environmentally sustainable behavior. The results showed that participants who had higher self-efficacy for sustainability behavior and a high belief in their variability of sustainability behavior had a higher motivation to attempt sustainability behavior, and they also reported more of this actual behavior. 

In our study, we used the SDGPI measure developed by Di Fabio and Rosen [30], to find data for SE development, as the data obtained through the SDGPI provide an opportunity for universities to support sustainable development in their operations. We examined college students’ attitude, interest, motivation, and self-efficacy with regard to 17 SDGs, and further identified through SDGPI how the psychological characteristics of college students are reflected in understanding next SDGs: environmental sustainability, health, and equity. First, to better understand college students’ self-perceptions of the SDGs, the ranking of importance of SDGs will be identified. Second, whether and how attitude, interest, motivation, and self-efficacy are correlated will be examined and investigated carefully. Third, the intercorrelations among attitude, interest, motivation, and self-efficacy across demographic groups (gender, grade, and major) will be examined (File S1); Fourth, whether attitude accounts for a proportion of the variance of interest, motivation, and self-efficacy will be investigated thoroughly; Fifth, whether attitude, interest, and motivation account for a proportion of the variance in self-efficacy will be explored.

## 3. Materials and Methods

### 3.1. Participants

All the participants were college students at a university in eastern China. Participants were recruited using convenience sampling procedures on campus (e.g., library, study room, and dormitory). A total of 368 students participated in the research, with an effective sample of 360 students (244 females, 116 males) and an invalid sample of 8 students with missing data. Among these 360 students, 20.6% (74) were freshmen, 29.7% (107) were sophomores, 34.7% (125) were juniors, 4.7% (17) were seniors, and 10.3% (37) were graduate students (Table 1).

### 3.2. The Sustainable Development Goals Psychological Inventory

Based on the SDGPI of Di Fabio and Rosen [30], our SDGPI is composed of 68 items. Each of the four subscales has 17 items, and the 17 items for each subscale correspond to the 17 SDGs (File S2). The participants were asked to rank (1) their attitude toward each SDG, (2) their interest in each SDG, (3) their motivation toward achieving each of the SDGs, and (4) their self-efficacy toward taking practical action to achieve the SDGs. The college students used a five-point Likert scale to rate the 68 SDGPI items (1 = not at all, 2 = a little, 3 = somewhat, 4 = quite a lot, and 5 = very much).

### 3.3. Data Analysis

To explore ways to develop SE to support students’ understanding of health, equity, and environmental sustainability, we examined East Chinese college students’ psychological characteristics including attitude, interests, motivations, and self-efficacy, in relation to the SDGs by using the SDGPI. First, we investigated college students’ current self-perception of the SDGs by asking them to rank the importance of the SDGs. Second, we estimated the reliability of the Chinese version of the SDGPI. Third, we accounted for the relationships of four psychological characteristics using the Mann-Whitney U test, Kruskal-Wallis one-way analysis, and regression analysis. Simple regression analysis was used to determine the proportion of variability between attitude, interest, motivation, and self-efficacy. This measure allows the researcher to identify which of the independent variables correlates with the dependent variable and to what degree. Multiple regression analysis was used for self-efficacy as there is more than one independent variable (e.g., attitude, interest, and motivation). All data were analyzed using the statistics software package SPSS version 26 (IBM Corp, Armonk, NY, USA).

### 3.4. Ethics Statement

According to the guidelines and regulations of Zhejiang Agriculture and Forestry University, this research did not require ethics approval. However, an ethical approach is to be expected. For this research, oral consent of the participants was obtained in accordance with the principles expressed in the Declaration of Helsinki. The names of the participants were not used to avoid their identification and the data were analyzed anonymously.

## 4. Results

### 4.1. The Most and Least Important SDGs

To investigate the importance of the SDGs, college students at Zhejiang A & F University in Zhejiang Province in eastern China were asked the following questions: (1) Which of the 17 SDGs do you think is the most important? (2) Which of the 17 SDGs do you think is the least important?

The top three most important SDGs based on the students’ answers were SDG 3 (good health and well-being) (49.72%), SDG 4 (quality education) (41.39%), and SDG 1 (no poverty) (32.22%) (Table 2). The top three least important SDGs were SDG 8 (decent work and economic growth) (41.11%), SDG 17 (partnerships for the goals) (38.06%), and SDG 12 (response consumption and institutions) (30.83%) (Table 3).

### 4.2. Reliability of Measurement Method

Data were collected using the SDGPI, which is classified into four subscales, named, as Attitude, Interest, Motivation, and Self-efficacy. In this work, Cronbach’s alpha [60] was used to evaluate the reliability of the SDGPI. Table 4 shows that Cronbach’s alpha coefficients were 0.831 for SDGs attitude, 0.860 for SDGs interest, 0.890 for SDGs motivation, and 0.829 for SDGs self-efficacy. The coefficient of the general method was 0.942. The reliability coefficients of all the SDGs dimensions exceeded 0.800; therefore, the dimensions can be considered very reliable. 

### 4.3. Sustainable Development Goals Psychological Inventory

The mean scores and standard deviations (SD) of the SDGPI are shown in Table 5. The results suggested that these means are homogenous.

### 4.4. Statistical Analysis

#### 4.4.1. Intercorrelations among SDGs Attitude, Interest, Motivation and Self-Efficacy

To examine whether there were significant correlations among all the variables of attitude, interest, motivation, and self-efficacy in the college student sample when examining sustainability behavior, Pearson product-moment correlation coefficients were computed to assess the relationships between the variables, as reported through the SDGs Attitude, SDGs Interest, SDGs Motivation, and SDGs Self-efficacy scores. The intercorrelations among factors can be seen in Table 6. The results show that attitude, interest, motivation, and self-efficacy were correlated with one another in relation to the college students.

#### 4.4.2. Mann-Whitney U Test and Kruskal-Wallis One-Way Test

The Mann-Whitney U test [61] was used to analyze participants’ gender differences, and the Kruskal-Wallis one-way [62] for grade and major differences. 

Table 7 shows that there were significant differences between men and women with respect to attitude, interest, and motivation, and women scored much higher on these three subscales than men. In contrast, there was no statistically significant difference in self-efficacy. Table 8 shows that there were no significant differences in grade with respect to attitude, interest, motivation, and self-efficacy. Table 9 shows that there were no significant differences in major with respect to attitude, interest, motivation, and self-efficacy.

To further understand the gender differences among the four subscales of the SDGPI, independent-sample *t*-tests were used and the results are listed in Table 10. The table shows the mean, SD, Levene’s test of equality of variance, and results of SDGs by gender, which indicates significant differences in both SDGs attitude and SDGs interest between men and women (*p* < 0.001) and significant differences in SDGs motivation (*p* < 0.01).

#### 4.4.3. Regression Analysis for SDGs Attitude Predicting SDGs Interest, Motivation and Self-Efficacy

The research used three simple regressions. The results revealed that attitude explained significant variance in interest, motivation, and self-efficacy. Table 11 shows the statistical significance for the regression of attitude on interest. The results for the regression of attitude on motivation are shown in Table 12. Table 13 indicates significance for the regression of attitude on self-efficacy. 

#### 4.4.4. Regression Analysis for SDGs Attitude, Interest, and Motivation Predicting Self-Efficacy

Multiple regression was used to determine the influence of attitude, interest, and motivation on self-efficacy. Table 14 indicates the significance of the regression of attitude, interest, and motivation on self-efficacy. 

## 5. Discussion

This study aimed to investigate the attitude, interest, motivation, and self-efficacy of Chinese college students with regard to the 17 SDGs. Demographic variables (gender, grade, and major) were also examined. 

### 5.1. Status Quo of College Students’ Self-Perceptions of the SDGs in Zhejiang Province in China

With respect to the question, “Which of the 17 SDGs do you think is the most important?” (Table 1), the college students particularly stressed SDG 3, “good health and well-being” (49.72%); SDG 4, “quality education” (41.39%); and SDG 1, “no poverty” (32.22%). Consistent with previous research [30], SDG 3, SDG 4, and SDG 1 were the top three most important SDGs. SDG 3 is dedicated to health and well-being for everyone at all ages [63,64], and it is also recognized as critical for achieving the other SDGs [65]. The results in the present work indicate that the realization of SDG 3 is very important to other SDGs, and the realization of other SDGs can be interpreted as contributing to the SDG 3. Because we analyzed a sample of college students from Zhejiang A & F University in Zhejiang Province in eastern China, our conclusions may not be extrapolated to other situations. However, health is a common interest of all of people worldwide and is given particular attention because of the severe threats from serious pandemics, such as COVID-19. Global warming caused by climate change can also accelerate the spread of some infectious diseases; thus, SDG 13 is linked to SDG 3. SDG 3 was also considered to be most important in the Eastern Mediterranean region, Africa, and Southeast Asia [66]. With respect to the question, “Which of the 17 SDGs do you think is the least important?” (Table 2), the college students identified SDG 8, “decent work and economic growth (41.11%); SDG 17, “partnerships for the goals” (38.06%); and SDG 12 “responsible consumption and institutions” (30.83%). The result supports a previous study [30] on SDG 17, but differs in that the other least important in that study were SDG 14 and SDG 0 (all important). Some SDGs (e.g., SDG 8 and SDG 17) may not be directly related to the lives of college students and were therefore ranked as least important. In addition, to establish stronger conclusions, it would be necessary to increase the sample size in the future, including a large number of college students from several Chinese universities. 

**Figure 2:**
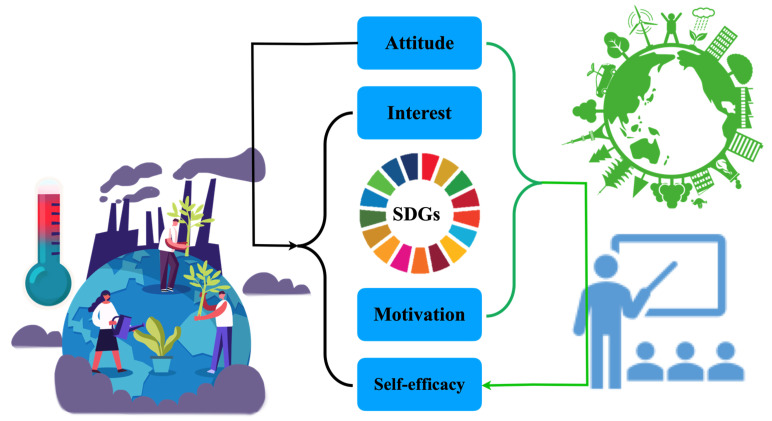
Scheme for the reflection of the characteristics of college students in understanding SDGs to improve personal performance and achieve sustainable development (own creation).

In support of university students’ holistic understanding of the SDGs, the focus on education for sustainable development goals (ESDG) has become a debate in social, environmental, and ecological justice [67]. According to Chaleta et al. [68], when the students were asked to profoundly rethink the three pillars of sustainable development and to link these pillars to ecological justice or biospheric egalitarianism, the most mentioned objectives were those related to SDG 4 (quality education), SDG 5 (gender equality), SDG 10 (reduced inequalities), SDG 8 (decent work and economic growth), and SDG 16 (peace, justice, and strong institutions). García-González et al. in their part have shown that significant changes were found in the students’ knowledge of the SDGs after participation in a training process on education for sustainability [69]. Consequently, education can be regarded as a necessary pillar for social transformation toward sustainable development in the current unsustainable environment [70] (Figure 2). In Figure 2, the black arrow indicates that SDGs attitude is a significant predictor of interest, motivation, and self-efficacy. The green arrow indicates that SDGs attitude, interest, and motivation can be used to predict self-efficacy.

### 5.2. SDGs Attitude, Interest, Motivation and Self-Efficacy Are All Significantly Positively Correlated 

Consistent with the existing literature on the variables of attitude, interest, motivation and self-efficacy, the results of this study revealed that these variables were all significantly positively correlated. Previous research has found significant positive correlations among interest, motivation, and self-efficacy [30]. Attitudes predict behavior and are considered the crown jewel of social psychology [71,72]. Research findings have also indicated that there is a significant relationship among interest, motivation, and a collaborative attitude toward academic performance [73]. Attitude toward mathematics is the most important factor explaining individual students’ academic achievement, which can be explained by the difference between students’ achievement motivation and perceived self-efficacy [74]. The findings in this work based on Bandura’s [75] theory of self-efficacy and Fabio’s [30] SDGPI also emphasized the benefits of using the psychology of sustainability to examine psychological issues that have been traditionally viewed through a deficit lens. Because sustainability development demands changes in human behavior [76], to an extent, the analysis of individual motives and values can be critical to providing a solution to unsustainability problems [77].

There is a relationship between motivation and interest in entrepreneurship [78], and self-motivation through proximal goal setting acts as an effective mechanism to cultivate competencies, self-efficacy, and intrinsic interest [75]. Biocentric nature values, human-centered values, pro-environmental and pro-social attitudes, interests, and motivations were found to be interrelated. Attitudes, interests, and motivations are negatively correlated with dismissive human and utilitarian nature values [79]. Researchers in China have explored how the public comprehends and forms supportive attitudes toward the SDGs [80]. The results indicated that there is an interaction effect between value orientations and knowledge in the public with regard to support for the SDGs (Figure 2), and the Chinese public regards the implementation of the SDGs as a part of development policy rather than environmental policy. 

### 5.3. Significant Differences between Men and Women with Respect to Attitude, Interest, and Motivation

One of the most notable findings within this study was the difference in attitude, interest, and motivation levels between male and female college students. The findings were consistent with previous findings [45,81] that revealed that the scores of motivation and attitude of female students were evidently higher than those of male students. This difference may be related to the stronger sense of social responsibility among females than among males, while social responsibility positively influences pro-environmental behavior [82]. Similarly, the countries with higher femininity orientation provided a higher quantity of sustainability reports [83]. Additionally, it was found that students’ technological self-efficacy is related to their attitude toward technology-based self-directed learning [84]. 

### 5.4. Attitude Accounts for a Significant Proportion of the Variance of Interest, Motivation, and Self-Efficacy

The results of this study revealed that attitude accounted for a significant proportion of the variance of interest, motivation, and self-efficacy (Figure 2). There is a negative significant correlation between students’ motivation for instrumental education, their instrumental performance self-efficacy beliefs, attitudes, and burnout [85]. Teachers’ attitudes, motivation, and self-efficacy are aspects of professional competence that affect students’ motivation and learning via instructional behavior [86]. Sociability, shyness, attitudes, motivation, and self-efficacy can predict social networking site (SNS) use. Sociability, attitudes, entertainment motivation, social interaction motivation, and self-efficacy are significant predictors of the social function of SNSs [87].

### 5.5. Significance of the Regression of Attitude, Interest, and Motivation on Self-Efficacy

Finally, in an attempt to identify variables that account for a significant proportion of variance in self-efficacy, a multiple regression analysis was conducted. The results indicated that attitude, interest, and motivation could all account for self-efficacy. Declines in interest/motivation/attitude were related to many variables, including self-efficacy [88]. Self-efficacy had a direct influence on intentions, and self-efficacy theory can be presented as a probable general model of attitude change [89]. 

## 6. Conclusions

To support sustainable development in higher education and foster college students’ understanding of the SDGs, in Zhejiang Province in eastern China, students’ psychological characteristics (attitudes, interest, motivation, and self-efficacy) in relation to the SDGs were identified and analyzed using the SDGPI. The results indicated that the top three most important SDGs for college students in China are SDG 3 (good health and well-being), SDG 4 (quality education), and SDG 1 (no poverty). In addition, there are significant relationships between attitude, interest, motivation, and self-efficacy with regard to the SDGs. Attitude was a significant predictor of interest, motivation, and self-efficacy, while attitude, interest and motivation accounted for self-efficacy. 

Education, research, innovation, and funding are key factors in achieving the SDGs [66]. Meanwhile, public health is not only the enabler, but also the major outcome of sustainable development [63]. Health (SDG 3) is a common goal for people around the world, and climate action (SDG 13), e.g., preventing human-caused global warming, can reduce the spread of some infectious diseases. Moreover, self-efficacy is associated with better satisfaction with life [90], and it also plays an important role in maintaining the psychological state of the people under stress [91] and directing health-related interventions [92]. In general, the analysis we conducted is a step on the journey toward raising college students’ awareness of the SDGs and protection of the environment, which will also improve public health by reconnecting people with nature to promote sustainability. 

Since the COVID-19 pandemic has interrupted the UN’s flagship plan to end poverty and protect the environment [93], it is necessary to end the crisis and to return to a pathway to environmental sustainability, health, and equity. This study shows that much remains to be done to promote SDGs at the university level. University education for sustainable development can increase students’ awareness of sustainable development in the following ways:

(a) By promoting college students’ self-perceptions of the SDGs and the importance of sustainability;
(b) By supporting the positive attitude of college students towards the SDGs e.g., through projected-based learning, case teaching, and collaborative learning;
(c) By stimulating college students’ SDGs interest and SDGs motivation by means of games and other group learning activities, and considering their needs when designing projects;
(d) By enhancing college students’ SDGs self-efficacy, and then stimulating their environmentally sustainable behavior and pro-environmental behavior.

This study provides a contribution to the psychology of sustainability with respect to the identification of college students on matters related to the SDGs. It is believed that the implementation of education for sustainability in higher education could support environmental sustainability. In the future, it would seem sensible to establish sustainable development curricula and training programs on the SDGs for college students so that they can take advantage of university resources on matters related to the SDGs. The promotion of college students’ sustainability consciousness affects environmental sustainability and further affects public health. 

## Figures and Tables

**Table 1:** Summary of the demographic statistics (N = 360).

Measure	Frequency	%
Gender		
male students	116	32.2
female students	244	67.8
Grade		
freshmen	74	20.6
sophomores	107	29.7
juniors	125	34.8
seniors	17	4.7
graduate students	37	10.2
Major		
humanities	168	46.7
science	104	28.9
engineering	88	24.4

**Table 2:** The most important SDGs.

Selected Items	(%)	Rank
Which of the 17 SDGs do you think is the most important? (Choose three items)		
1. No poverty	32.22	3
2. Zero hunger	30.00	4
3. Good health and well-being	49.72	1
4. Quality education	41.39	2
5. Gender equality	28.33	5
6. Clean water and sanitation	15.00	8
7. Affordable and clean energy	13.61	9
8. Decent work and economic growth	10.00	12
9. Industry, innovation, and infrastructure	12.78	10
10. Reduced inequalities	16.39	6
11. Sustainable cities and communities	15.56	7
12. Response consumption and institutions	4.44	15
13. Climate action	8.33	13
14. Life below water	3.06	16
15. Life on land	1.67	17
16. Peace, justice, and strong institutions	11.67	11
17. Partnerships for the goals	5.83	14

Note: N = 360.

**Table 3:** The least important SDGs.

Selected Items	(%)	Rank
Which of the 17 SDGs do you think is the least important? (Choose three items)		
1. No poverty	26.11	4
2. Zero hunger	23.06	5
3. Good health and well-being	6.67	16
4. Quality education	7.22	15
5. Gender equality	9.44	14
6. Clean water and sanitation	4.17	17
7. Affordable and clean energy	11.67	11
8. Decent work and economic growth	41.11	1
9. Industry, innovation, and infrastructure	13.61	9
10. Reduced inequalities	11.11	12
11. Sustainable cities and communities	12.22	10
12. Response consumption and institutions	30.83	3
13. Climate action	11.11	12
14. Life below water	17.5	7
15. Life on land	14.17	8
16. Peace, justice, and strong institutions	21.94	6
17. Partnerships for the goals	38.06	2

Note: N = 360.

**Table 4:** Reliability estimates for SDGPI.

Factors	Items	Cronbach’s Alpha
SDGs Attitude	1, 2, 3, 4, 5, 6, 7, 8, 9, 10, 11, 12, 13, 14, 15, 16, 17	0.831
SDGs Interest	18, 19, 20, 21, 22, 23, 24, 25, 26, 27, 28, 29, 30, 31, 32, 33, 34	0.860
SDGs Motivation	35, 36, 37, 38, 39, 40, 41, 42, 43, 44, 45, 46, 47, 48, 49, 50, 51	0.890
SDGs Self-efficacy	52, 53, 54, 55, 56, 57, 58, 59, 60, 61, 62, 63, 64, 65, 66, 67, 68	0.829

Note: N = 360.

**Table 5:** Means and standard deviations for each dimension.

Measure	M	SD	Minimum	Maximum
SDGs Attitude	69.67	9.21	30	85
SDGs Interest	61.86	8.82	30	85
SDGs Motivation	63.50	9.56	32	85
SDGs Self-efficacy	55.40	7.86	26	85

Note: N = 360. Scale ranged from 1–5.

**Table 6:** Matrix of intercorrelations among factors.

Factor	SDGs Attitude	SDGs Interest	SDGs Motivation	SDGs Self-Efficacy
SDGs Attitude	_			
SDGs Interest	0.635 **	_		
SDGs Motivation	0.655 **	0.774 **	_	
SDGs Self-efficacy	0.254 **	0.517 **	0.565 **	_

Note: N = 360 ** = *p* < 0.01.

**Table 7:** Mann-Whitney U test for gender differences.

Variable	SDGs Attitude	SDGs Interest	SDGs Motivation	SDGs Self-Efficacy
Mann-Whitney U	18,793.500	16,695.500	17,185.500	13,411.500
Wilcoxon W	48,683.500	46,585.500	47,075.500	43,301.500
Z	5.003	2.579	3.290	−0.804
Asymp. Sig. (2-tailed)	0.000	0.006	0.001	0.421

**Table 8:** Kruskal-Wallis test for grade differences.

Variable	SDGs Attitude	SDGs Interest	SDGs Motivation	SDGs Self-Efficacy
Chi-Square	8.738	6.796	8.437	9.259
Df	4	4	4	4
Asymp. Sig.	0.068	0.137	0.080	0.055

**Table 9:** Kruskal-Wallis test for major differences.

Variable	SDGs Attitude	SDGs Interest	SDGs Motivation	SDGs Self-Efficacy
Chi-Square	0.014	0.484	0.486	0.893
Df	2	2	2	2
Asymp. Sig.	0.993	0.785	0.784	0.640

**Table 10:** Differences among the four subscales of SDGPI by gender.

Variable	Men		Women		Sig of Levene’s Test	t
	M	SD	M	SD		
SDGs Attitude	66.46	9.01	71.20	8.92	0.842	−4.70 ***
SDGs Interest	60.07	9.05	62.71	8.59	0.434	−2.68 **
SDGs Motivation	61.28	9.82	64.56	9.26	0.352	−3.07 **
SDGs Self-efficacy	55.76	7.95	55.23	7.82	0.928	0.592

Note: N = 360 ** = *p* < 0.01, *** *p* < 0.001.

**Table 11:** Summary of regression analysis for SDGs attitude predicting SDGs interest.

Variable	B	SE B	B	Sig
(Constant)	19.499	2.749	0.000	
SDGs Attitude	0.608	0.039	0.635	0.000
*R* ^2^	0.403			
Sig. of regression	0.635			

**Table 12:** Summary of regression analysis for SDGs attitude predicting SDGs motivation.

Variable	B	SE B	B	Sig
(Constant)	16.178	2.914	0.000	
SDGs Attitude	0.679	0.041	0.655	0.000
*R* ^2^	0.403			
Sig. of regression	0.655			

**Table 13:** Summary of regression analysis for SDGs attitude predicting SDGs self-efficacy.

Variable	B	SE B	B	Sig
(Constant)	40.285	3.064	0.000	
SDGs Attitude	0.217	0.044	0.254	0.000
*R* ^2^	0.065			
Sig. of regression	0.254			

**Table 14:** Summary of regression analysis for SDGs attitude, interest, and motivation predicting self-efficacy.

Variable	B	SE B	B	Sig
(Constant)	28.458	2.696	0.000	
SDGs Attitude	−0.226	0.049	−0.265	0.000
SDGs Interest	0.252	0.061	0.283	0.000
SDGs Motivation	0.427	0.058	0.519	0.000
*R* ^2^	0.372			
Sig. of regression	0.000			

## Data Availability

Not applicable.

## Supplementary Materials

The following are available online at https://www.mdpi.com/article/10.3390/ijerph18158217/s1, File S1: Demographic Questionnaire, File S2: The Sustainable Development Goals Psychological Inventory.

## References

[1] Jeronen E., Idowu S.O., Capaldi N., Das Gupta A., Zu L. (2013). Sustainability and Sustainable Development. Encyclopedia of Corporate Social Responsibility.

[2] Jeronen E., Idowu S., Schmidpeter R., Capaldi N., Zu L., del Baldo M., Abreu R. (2020). Sustainable Development. Encyclopedia of Sustainable Management.

[3] UN (2015). Transforming Our World: The 2030 Agenda for Sustainable Development.

[4] Tikkinen K.A.O., Malekzadeh R., Schlegel M., Rutanen J., Glasziou P. (2020). COVID-19 clinical trials: Learning from exceptions in the research chaos. Nat. Med..

[5] Pan S.L., Zhang S. (2020). From fighting COVID-19 pandemic to tackling sustainable development goals: An opportunity for responsible information systems research. Int. J. Inf. Manag..

[6] Leal Filho W., Azul A.M., Wall T., Vasconcelos C.R.P., Salvia A.L., do Paco A., Shulla K., Levesque V., Doni F., Alvarez-Castanon L. (2020). COVID-19: The impact of a global crisis on sustainable development research. Sustain. Sci..

[7] Leal Filho W. (2020). COVID-19, sustainable development and higher education: Towards a recovery path. Int. J. Sustain. High. Educ..

[8] Liu S. (2020). Higher education and Sustainable Developement Goals during COVID-19: Coping strategies of a university in Wuhan, China. J. Public Health Res..

[9] Mejia R., Hotez P., Bottazzi M.E. (2020). Global COVID-19 Efforts as the Platform to Achieving the Sustainable Development Goals. Curr. Trop. Med. Rep..

[10] Leal Filho W., Brandli L.L., Lange Salvia A., Rayman-Bacchus L., Platje J. (2020). COVID-19 and the UN Sustainable Development Goals: Threat to Solidarity or an Opportunity?. Sustainability.

[11] The Lancet Public Health (2020). Will the COVID-19 pandemic threaten the SDGs?. Lancet Public Health.

[12] Nundy S., Ghosh A., Mesloub A., Albaqawy G.A., Alnaim M.M. (2021). Impact of COVID-19 pandemic on socio-economic, energy-environment and transport sector globally and sustainable development goal (SDG). J. Clean. Prod..

[13] Safitri Y., Ningsih R.D., Agustianingsih D.P., Sukhwani V., Kato A., Shaw R. (2021). COVID-19 Impact on SDGs and the Fiscal Measures: Case of Indonesia. Int. J. Environ. Res. Public Health.

[14] Adhikari J., Timsina J., Khadka S.R., Ghale Y., Ojha H. (2021). COVID-19 impacts on agriculture and food systems in Nepal: Implications for SDGs. Agric. Syst..

[15] Bherwani H., Gautam S., Gupta A. (2021). Qualitative and quantitative analyses of impact of COVID-19 on sustainable development goals (SDGs) in Indian subcontinent with a focus on air quality. Int. J. Environ. Sci. Technol. (Tehran).

[16] Da Silva F.R., Câmara S.F., Pinto F.R., Soares M., Viana M.B., De Paula T.M. (2021). Sustainable Development Goals Against Covid-19: The Performance Of Brazilian Cities In Sdgs 3 And 6 And Their Reflection On The Pandemic. Geogr. Environ. Sustain..

[17] Paital B., Das K., Parida S.K. (2020). Inter nation social lockdown versus medical care against COVID-19, a mild environmental insight with special reference to India. Sci. Total Environ..

[18] Anholon R., Rampasso I.S., Martins V.W.B., Serafim M.P., Leal Filho W., Quelhas O.L.G. (2021). COVID-19 and the targets of SDG 8: Reflections on Brazilian scenario. Kybernetes.

[19] Chabbott C., Sinclair M. (2020). SDG 4 and the COVID-19 emergency: Textbooks, tutoring, and teachers. Prospects (Paris).

[20] Yunus A.P., Masago Y., Hijioka Y. (2020). COVID-19 and surface water quality: Improved lake water quality during the lockdown. Sci. Total Environ..

[21] Sterling S. (2010). Learning for resilience, or the resilient learner? Towards a necessary reconciliation in a paradigm of sustainable education. Environ. Educ. Res..

[22] Barth M. (2016). Teaching and Learning in Sustainability Science. Sustainability Science.

[23] Klassen R.M., Tze V.M.C. (2014). Teachers’ self-efficacy, personality, and teaching effectiveness: A meta-analysis. Educ. Res. Rev..

[24] Bardach L., Klassen R.M., Perry N.E. (2021). Teachers’ Psychological Characteristics: Do They Matter for Teacher Effectiveness, Teachers’ Well-being, Retention, and Interpersonal Relations? An Integrative Review. Educ. Psychol. Rev..

[25] Pintrich P.R., Groot E.V.D. (1990). Motivational and Self-Regulated Learning Components of Classroom Academic Performance. J. Educ. Psychol..

[26] Kaiser F.G., Wolfing S.F., Fuhrer U. (1999). Environmental attitude and ecological behavior. J. Environ. Psychol..

[27] Yu T., Lei Z., Dezhi S. (2006). Functions and behaviors of activated sludge extracellular polymeric substances (EPS): A promising environmental interest. J. Environ. Sci..

[28] Bockarjova M., Steg L. (2014). Can Protection Motivation Theory predict pro-environmental behavior? Explaining the adoption of electric vehicles in the Netherlands. Glob. Environ. Chang..

[29] Tabernero C., Hernández B. (2010). Self-Efficacy and Intrinsic Motivation Guiding Environmental Behavior. Environ. Behav..

[30] Di Fabio A., Rosen M.A. (2020). An Exploratory Study of a New Psychological Instrument for Evaluating Sustainability: The Sustainable Development Goals Psychological Inventory. Sustainability.

[31] Van der Vliet N., Staatsen B., Kruize H., Morris G., Costongs C., Bell R., Marques S., Taylor T., Quiroga S., Martinez Juarez P. (2018). The INHERIT Model: A Tool to Jointly Improve Health, Environmental Sustainability and Health Equity through Behavior and Lifestyle Change. Int. J. Environ. Res. Public Health.

[32] Stegeman I., Godfrey A., Romeo-Velilla M., Bell R., Staatsen B., van der Vliet N., Kruize H., Morris G., Taylor T., Strube R. (2020). Encouraging and Enabling Lifestyles and Behaviours to Simultaneously Promote Environmental Sustainability, Health and Equity: Key Policy Messages from INHERIT. Int. J. Environ. Res. Public Health.

[33] Kay V.A., Livingstone C.H. (2020). Promoting environmental sustainability, equity and health in Victorian Primary Care Partnerships. Health Promot. J. Aust..

[34] Oskamp S. (2000). A sustainable future for humanity? How can psychology help?. Am. Psychol..

[35] Jaipal R. (2017). Psychology at the Crossroads. Psychol. Dev. Soc..

[36] Raymond I.J., Raymond C.M. (2019). Positive psychology perspectives on social values and their application to intentionally delivered sustainability interventions. Sustain. Sci..

[37] Di Fabio A., Rosen M.A. (2018). Opening the Black Box of Psychological Processes in the Science of Sustainable Development: A New Frontier. Eur. J. Sustain. Dev. Res..

[38] Di Fabio A. (2017). The Psychology of Sustainability and Sustainable Development for Well-Being in Organizations. Front. Psychol..

[39] Di Fabio A. (2017). Positive Healthy Organizations: Promoting Well-Being, Meaningfulness, and Sustainability in Organizations. Front. Psychol..

[40] Manuti A., Giancaspro M. (2019). People Make the Difference: An Explorative Study on the Relationship between Organizational Practices, Employees’ Resources, and Organizational Behavior Enhancing the Psychology of Sustainability and Sustainable Development. Sustainability.

[41] Brundtland G.H. (2009). Our Common Future—Call for Action. Environ. Conserv..

[42] Organisation for Economic Cooperation and Development (2010). PISA 2009 Assessment Framework: Key Competencies in Reading, Mathematics and Science.

[43] Glasman L.R., Albarracin D. (2006). Forming attitudes that predict future behavior: A meta-analysis of the attitude-behavior relation. Psychol. Bull..

[44] Salas-Zapata W., Cardona-Arias J.A. (2020). Knowledge, attitudes and practices of sustainability in two university populations, Colombia. J. Appl. Res. High. Ed..

[45] Al-Naqbi A.K., Alshannag Q. (2018). The status of education for sustainable development and sustainability knowledge, attitudes, and behaviors of UAE University students. Int. J. Sust. High. Ed..

[46] Leiserowitz A.A., Kates R.W., Parris T.M. (2006). Sustainability Values, Attitudes, and Behaviors: A Review of Multinational and Global Trends. Annu. Rev. Environ. Resour..

[47] Wang H.-H., Hong Z.-R., Lin H.-S., Tsai C.-Y. (2020). The relationships among adult sustainability attitudes, psychological well-being, nature relatedness, and interest in scientific issues. Curr. Psychol..

[48] Misseyanni A., Marouli C., Papadopoulou P. (2020). How Teaching Affects Student Attitudes towards the Environment and Sustainability in Higher Education: An Instructors’ Perspective. Eur. J. Sustain. Dev..

[49] Probst L., Bardach L., Kamusingize D., Templer N., Ogwali H., Owamani A., Mulumba L., Onwonga R., Adugna B.T. (2019). A transformative university learning experience contributes to sustainability attitudes, skills and agency. J. Clean. Prod..

[50] Smaniotto C., Battistella C., Brunelli L., Ruscio E., Agodi A., Auxilia F., Baccolini V., Gelatti U., Odone A., Prato R. (2020). Sustainable Development Goals and 2030 Agenda: Awareness, Knowledge and Attitudes in Nine Italian Universities, 2019. Int. J. Environ. Res. Public Health.

[51] Tang K.H.D. (2018). Correlation between sustainability education and engineering students’ attitudes towards sustainability. Int. J. Sustain. High. Ed..

[52] Nousheen A., Yousuf Zai S.A., Waseem M., Khan S.A. (2020). Education for sustainable development (ESD): Effects of sustainability education on pre-service teachers’ attitude towards sustainable development (SD). J. Clean. Prod..

[53] Lambin E.F. (2005). Conditions for sustainability of human—Environment systems: Information, motivation, and capacity. Glob. Environ. Chang..

[54] Hansen D.K. (2014). Understanding Motivations for Modern Sustainability. Master’s Thesis.

[55] Schiefele U., Schaffner E. (2015). Teacher interests, mastery goals, and self-efficacy as predictors of instructional practices and student motivation. Contemp. Educ. Psychol..

[56] Surjanti J., Soejoto A., Seno D.N., Waspodo (2020). Mangrove forest ecotourism: Participatory ecological learning and sustainability of students’ behavior through self-efficacy and self-concept. Soc. Sci. Humanit. Open.

[57] Kornilaki M., Thomas R., Font X. (2019). The sustainability behaviour of small firms in tourism: The role of self-efficacy and contextual constraints. J. Sustain. Tour..

[58] Perrault E.K., Clark S.K. (2018). Sustainability attitudes and behavioral motivations of college students. Int. J. Sustain. High. Ed..

[59] Schutte N.S., Bhullar N. (2017). Approaching Environmental Sustainability: Perceptions of Self-Efficacy and Changeability. J. Psychol..

[60] Tavakol M., Dennick R. (2011). Making sense of Cronbach’s alpha. Int. J. Med. Educ..

[61] Mann H.B., Whitney D.R. (1947). On a test of whether one of two random variables is stochastically larger than the other. Ann. Math. Stat..

[62] Kruskal W.H., Wallis W.A. (1952). Use of ranks in one-criterion variance analysis. J. Am. Stat. Assoc..

[63] Menne B., Aragon de Leon E., Bekker M., Mirzikashvili N., Morton S., Shriwise A., Tomson G., Vracko P., Wippel C. (2020). Health and well-being for all: An approach to accelerating progress to achieve the Sustainable Development Goals (SDGs) in countries in the WHO European Region. Eur. J. Public Health.

[64] Skevington S.M., Epton T. (2018). How will the sustainable development goals deliver changes in well-being? A systematic review and meta-analysis to investigate whether WHOQOL-BREF scores respond to change. BMJ Glob. Health.

[65] Nunes A.R., Lee K., O’Riordan T. (2016). The importance of an integrating framework for achieving the Sustainable Development Goals: The example of health and well-being. BMJ Glob. Health.

[66] Sweileh W.M. (2020). Bibliometric analysis of scientific publications on “sustainable development goals” with emphasis on “good health and well-being” goal (2015–2019). Glob. Health.

[67] Kopnina H. (2020). Education for Sustainable Development Goals (ESDG): What Is Wrong with ESDGs, and What Can We Do Better?. Educ. Sci..

[68] Chaleta E., Saraiva M., Leal F., Fialho I., Borralho A. (2021). Higher Education and Sustainable Development Goals (SDG)—Potential Contribution of the Undergraduate Courses of the School of Social Sciences of the University of Évora. Sustainability.

[69] García-González E., Jiménez-Fontana R., Azcárate P. (2020). Education for Sustainability and the Sustainable Development Goals: Pre-Service Teachers’ Perceptions and Knowledge. Sustainability.

[70] Agirreazkuenaga L. (2019). Embedding Sustainable Development Goals in Education. Teachers’ Perspective about Education for Sustainability in the Basque Autonomous Community. Sustainability.

[71] Crano W.D., Prislin R. (2006). Attitudes and persuasion. Annu. Rev. Psychol..

[72] Albarracin D., Shavitt S. (2018). Attitudes and Attitude Change. Annu. Rev. Psychol..

[73] Azis P.A. (2016). Hubungan minat, motivasi belajar dan sikap dengan hasil belajar siswa kelas viii smp negeri 13 makassar. J. EST.

[74] Damrongpanit S. (2019). From Modern Teaching to Mathematics Achievement: The Mediating Role of Mathematics Attitude, Achievement Motivation, and Self-Efficacy. Eur. J. Educ. Res..

[75] Bandura A., Schunk D.H. (1981). Cultivating Competence, Self-Efficacy, and Intrinsic Interest Through Proximal Self-Motivation. J. Pers. Soc. Psychol.

[76] Fischer J., Dyball R., Fazey I., Gross C., Dovers S., Ehrlich P.R., Brulle R.J., Christensen C., Borden R.J. (2012). Human behavior and sustainability. Front. Ecol. Environ..

[77] Ehrlich P.R., Kennedy D. (2005). Millennium Assessment of Human Behavior. Science.

[78] Aji A.D., Sofyandi H., Tarmidi D., Saefudin N. (2019). The Effect of Self-Efficacy, Creativity, and Motivation on Entrepreneurship Interest in FBM Students of Widyatama University, Indonesia. Glob. Bus. Manag. Res. Int. J..

[79] Uitto A., Saloranta S. (2010). The relationship between secondary school students’ environmental and human values, attitudes, interests and motivations. Procedia Soc. Behav. Sci..

[80] Guan T., Meng K., Liu W., Xue L. (2019). Public Attitudes toward Sustainable Development Goals: Evidence from Five Chinese Cities. Sustainability.

[81] Wesley J.W. (2002). A Study of Academic Achievement, Attitude, Motivation, General Self-Efficacy, and Selected Demographic Characteristics of Community College Students. Ph.D. Thesis.

[82] Ahmad N., Ullah Z., Mahmood A., Ariza-Montes A., Vega-Munoz A., Han H., Scholz M. (2021). Corporate Social Responsibility at the Micro-Level as a “New Organizational Value” for Sustainability: Are Females More Aligned towards It?. Int. J. Environ. Res. Public Health.

[83] Gallén M.L., Peraita C. (2017). The Relationship between Femininity and Sustainability Reporting. Corp. Soc. Responsib. Environ. Manag..

[84] Pan X. (2020). Technology Acceptance, Technological Self-Efficacy, and Attitude Toward Technology-Based Self-Directed Learning: Learning Motivation as a Mediator. Front. Psychol..

[85] Girgin D. (2020). Motivation, Self-Efficacy and Attitude as Predictors of Burnout in Musical Instrument Education in Fine Arts High Schools. Eurasian J. Educ. Res..

[86] Oerke B., McElvany N., Ohle-Peters A., Horz H., Ullrich M. (2018). Einstellungen, Motivation und Selbstwirksamkeit von Lehrkräften. Z. Erzieh..

[87] Wang J.-L., Jackson L.A., Wang H.-Z., Gaskin J. (2015). Predicting Social Networking Site (SNS) use: Personality, attitudes, motivation and Internet self-efficacy. Personal. Individ. Differ..

[88] Potvin P., Hasni A. (2014). Interest, motivation and attitude towards science and technology at K-12 levels: A systematic review of 12 years of educational research. Stud. Sci. Educ..

[89] Maddux J.E. (1983). Protection motivation and self-Efficacy: A revise theory of fear appeals and attitude change. J. Exp. Soc. Psychol..

[90] Galin S., Heruti I., Barak N., Gotkine M. (2018). Hope and self-efficacy are associated with better satisfaction with life in people with ALS. Amyotroph. Lateral Scler. Front. Degener..

[91] Ching Fan S., Shih H.-C., Tseng H.-C., Chang K.-F., Li W.-C., Shin Shia A. (2021). Self-Efficacy Triggers Psychological Appraisal Mechanism for Mindset Shift. Int. J. Ment. Health Promot..

[92] Samendinger S., Hill C.R., Hepler T.J., Feltz D.L. (2019). Why Residuals Are Important in the Self-Efficacy-Performance Relationship Analysis: A Study Across 12 Cycling Sessions. J. Phys. Act. Health.

[93] Editorials (2021). How science can put the Sustainable Development Goals back on track. Nature.

