# Supplementary material for: Toward Environmental Sustainability, Health, and Equity: How the Psychological Characteristics of College Students Are Reflected in Understanding Sustainable Development Goals
Source: Int J Environ Res Public Health. 2021 Aug 3;18(15):8217. doi: 10.3390/ijerph18158217 (PMC8346037; doi:10.3390/ijerph18158217)
Supplement: Supplementary file 1 [file ijerph-18-08217-s001.zip › ijerph-1295556-supplementary.pdf]

File S1. Demographic Questionnaire

1. Please indicate your biological sex:

a. Male_______ b. Female________

2. Please indicate your grade:

a. Freshmen_______ b. Sophomores_______ c. Juniors________ d. Seniors_______

e. Graduate students________

3. Please indicate your major:

a. Humanities b. Science c. Engineering

4. Which of the 17 sustainable development goals (SDGs) do you think is the most important? (Choose three items)_________

a. No poverty b. Zero hunger c. Good health and well-being d. Quality education

e. Gender equality f. Clean water and sanitation g. Affordable and clean energy

h. Decent work and economic growth i. Industry, innovation, and infrastructure

j. Reducing inequalities k. Sustainable cities and communities

l. Responsible consumption and production m. Climate action n. Life below water

o. Life on land p. Peace, justice, and strong institutions q. Partnerships for the goals

5. Which of the 17 sustainable development goals (SDGs) do you think is the least important? (Choose three items)__________

a. No poverty b. Zero hunger c. Good health and well-being d. Quality education

e. Gender equality f. Clean water and sanitation g. Affordable and clean energy

h. Decent work and economic growth i. Industry, innovation, and infrastructure

j. Reducing inequalities k. Sustainable cities and communities

l. Responsible consumption and production m. Climate action n. Life below water

o. Life on land p. Peace, justice, and strong institutions q. Partnerships for the goals

File S2. The Sustainable Development Goals Psychological Inventory

Instructions: The sustainable development goals (SDGs) are a set of 17 global goals agreed to by the United Nations General Assembly. Please indicate your rankings first for all the items in column A, then for those in column B and C, and finally for those in column D. There are no right or wrong answers. Please use the following rankings: 1 = not at all; 2 = a little; 3 = somewhat; 4 = quite a lot; 5 = very much.

The Sustainable Development Goals Psychological Inventory																				
Regarding each of the following 17 SDGs to what extent	A					B					C					D				
	I think it's necessary to realize					I'm interested in					I'm motivated to act concretely for					I feel able to act concretely for				
1. No poverty	1	2	3	4	5	1	2	3	4	5	1	2	3	4	5	1	2	3	4	5
2. Zero hunger	1	2	3	4	5	1	2	3	4	5	1	2	3	4	5	1	2	3	4	5
3. Good health and well-being	1	2	3	4	5	1	2	3	4	5	1	2	3	4	5	1	2	3	4	5
4. Quality education	1	2	3	4	5	1	2	3	4	5	1	2	3	4	5	1	2	3	4	5
5. Gender equality	1	2	3	4	5	1	2	3	4	5	1	2	3	4	5	1	2	3	4	5
6. Clean water and sanitation	1	2	3	4	5	1	2	3	4	5	1	2	3	4	5	1	2	3	4	5
7. Affordable and clean energy	1	2	3	4	5	1	2	3	4	5	1	2	3	4	5	1	2	3	4	5
8. Decent work and economic growth	1	2	3	4	5	1	2	3	4	5	1	2	3	4	5	1	2	3	4	5
9. Industry, innovation, and infrastructure	1	2	3	4	5	1	2	3	4	5	1	2	3	4	5	1	2	3	4	5
10. Reducing inequalities	1	2	3	4	5	1	2	3	4	5	1	2	3	4	5	1	2	3	4	5
11. Sustainable cities and communities	1	2	3	4	5	1	2	3	4	5	1	2	3	4	5	1	2	3	4	5
12. Responsible consumption and production	1	2	3	4	5	1	2	3	4	5	1	2	3	4	5	1	2	3	4	5
13. Climate action	1	2	3	4	5	1	2	3	4	5	1	2	3	4	5	1	2	3	4	5
14. Life below water	1	2	3	4	5	1	2	3	4	5	1	2	3	4	5	1	2	3	4	5
15. Life on land	1	2	3	4	5	1	2	3	4	5	1	2	3	4	5	1	2	3	4	5
16. Peace, justice, and strong institutions	1	2	3	4	5	1	2	3	4	5	1	2	3	4	5	1	2	3	4	5
17. Partnerships for the goals	1	2	3	4	5	1	2	3	4	5	1	2	3	4	5	1	2	3	4	5
